# Maintaining osmotic balance in legume nodules

**DOI:** 10.1093/jxb/erab425

**Published:** 2022-01-05

**Authors:** Philip S Poole, Raphael Ledermann

**Affiliations:** Department of Plant Sciences, University of Oxford, South Parks Road, Oxford OX1 3RB, UK

**Keywords:** Legumes, N_2_ fixation, nodulation, ononitol, osmotic balance, pinitol, reactive oxygen species

## Abstract

This article comments on:

**Tian L, Liu L, Xu S, Deng R, Wu P, Jiang H, Wu G, Chen Y.** 2022. A d-pinitol transporter, LjPLT11, regulates plant growth and nodule development in *Lotus japonicus*. Journal of Experimental Botany **73**, 351–365.

**Pinitol (3-*O*-methyl-d-chiro-inositol) is shown to be transported passively across the plant-derived symbiosome membrane in *Lotus japonicus* by the energy-independent polyol transporter LjPLT11. Accumulation of pinitol is crucial to maintenance of osmotic balance in nodules. RNAi lines reduced in LjPLT11 have disrupted symbiosome membranes, reduced N_2_ fixation by rhizobial bacteroids and reduced plant growth. There is also an increase in production of reactive oxygen species (ROS) in LjPLT11 RNAi lines, consistent with osmotic imbalance causing increased stress in nodules.**

Biological nitrogen fixation provides 50–70 Tg of bioavailable nitrogen in agricultural systems per year, and sustains global food security. The most efficient contribution to biologically fixed nitrogen is from symbioses between legumes and rhizobia (Herridge *et al.*, 2008), which are soil bacteria that induce formation of nodules on plant roots. Legumes initiate nodule formation by the release of flavonoids, which induce *nod* genes in rhizobia. In turn, the bacteria synthesize lipo-chito-oligosaccharides (LCOs), that are detected by plant LysM-type receptors. This leads to activation of the common Sym pathway that is shared with the more ancient mycorrhizal symbiosis (Oldroyd and Downie, 2004). Root hairs curl, entrapping rhizobia in an infection pocket. Plants form an infection tube which bacteria enter and move down, while remaining extracellular to host cells. At the same time as infection threads develop, nodule development proceeds in the plant cortex, with the developing nodule meeting the dividing and ramifying infections threads. It has recently been shown that lateral roots and nodules have overlapping developmental programmes, with *Nodule INception* activator (*NIN*) initiating the same programme and sharing downstream activators with the lateral root programme (Schiessl *et al.*, 2019; Soyano *et al.*, 2019). Eventually, rhizobia are endocytosed into the cytoplasm of nodule cells, where the N_2_-fixing form of rhizobia (bacteroids) develop and are surrounded by the bacterial cell- and plant-derived symbiosome membranes. Together, the plant membrane and bacteroids are known as symbiosomes and the space between the two membranes is the symbiosome space. Nodules are either determinate, as in beans, soybeans, and *L. japonicus*, or indeterminate, as in alfalfa, *Medicago truncatula*, pea, and clover. Determinate nodules have a transient meristem resulting in infected plant cells being at the same development stage, with nodules growing larger by cell expansion. Typically, these nodules have several bacteroids enclosed by a single symbiosome membrane all in the same developmental state. Indeterminate nodules have a persistent meristem with distinct development zones from the tip, which is distal to the root, to the base, which is proximal to the root. Indeterminate nodules usually have symbiosomes with a single bacteroid (Oldroyd *et al.*, 2011; Oldroyd and Downie, 2008; Poole and Udvardi, 2013). Distal zone I contains the nodule meristem, zone II branching infection threads, zone II/III interzone developing bacteroids, zone III mature N_2_-fixing symbiosomes, and zone IV senescing symbiosomes.

As bacteroids develop, large changes occur in the transcriptome and proteome, with N_2_ fixation genes induced, but most genes required for growth, including ribosomal proteins, DNA replication, and amino acid biosynthesis, having reduced transcription (Barnett *et al.*, 2004; Becker *et al.*, 2004; Karunakaran *et al.*, 2009; Pessi *et al.*, 2007). Some legumes, such as those in a phylogenetic group known as the Inverted Repeat-Lacking Clade (IRLC legumes) produce from seven to >700 nodule cysteine-rich (NCR) peptides (Mergaert *et al.*, 2003; Guefrachi *et al.*, 2014; Montiel *et al.*, 2017; Roy *et al.*, 2020). These can be found in many common agriculturally important legumes such as pea, although most work has been done on the model indeterminate legume *Medicago truncatula*, where the peptides control bacteroid development. The model determinate legume *L. japonicus* does not produce NCR peptides, so while they exert precise control of bacteroid development, resulting in profound changes in bacteroid physiology and possibly fixation efficiency, they are not essential for the development of an effective symbiosis.

The symbiosome is the effective N_2_-fixing engine of symbiosis, with the plant providing carbon in the form of dicarboxylic acids (mainly l-malate and succinate) as well as metals, and even a key ligand for the N_2_-reducing bacterial enzyme nitrogenase in the form of homocitrate to bacteroids. All nutrients have to traverse two membranes, the first of which is the plant-derived symbiosome membrane (also called the peribacteroid membrane), which is effectively inverted so that movement from the plant cytosol to the symbiosome space equates to export. Metabolites can accumulate in the symbiosome space depending on whether they are then transported across the bacteroid membrane and utilized in metabolism, or not. The main carbon sources, the dicarboxylic acids, are catabolized by the tricarboxylic acid (TCA) cycle. Recent modelling and ^13^C metabolic flux analysis showed that catabolism of dicarboxylates requires more oxygen but also produces a high (NADH/NAD^+^) ratio suited to N_2_ reduction. While a low O_2_ level is required to protect nitrogenase from inactivation, plants limit oxygen supply to bacteroids so as to restrict the decarboxylating arm of the TCA cycle, which limits ammonia assimilation into glutamate. Plants control O_2_ levels in nodules with an O_2_ diffusion barrier and the synthesis of O_2_ binding haem proteins, the leghaemoglobins (Ott *et al.*, 2005). Such a tight control of oxygen supply by legumes while providing dicarboxylates as the energy and electron source donors for N_2_ fixation, with their high O_2_ requirement for metabolism, promotes ammonia secretion rather than assimilation into the central amino acid glutamate by bacteroids (Schulte *et al.*, 2021). However, modelling, in agreement with experimental studies, also shows that as the O_2_ supply becomes even more limited, alanine as well as ammonia will be secreted by bacteroids. This is because alanine synthesis removes pyruvate and reductant (NADH) from the bacteroid and prevents further oxidative stress due to NADH synthesis by the TCA cycle.

While oxygen is clearly the limiting nutrient that controls much of nodule metabolism, osmotic balance is clearly critical to N_2_ fixation in nodules. Synthesis of cyclic β-glucan, which seems to balance osmotic potential in the bacterial periplasm (the space between the inner and outer bacterial membranes) by nodule bacteria is essential for effective N_2_-fixing nodules (Bhagwat *et al.*, 1999). In the study reported by Tian *et al.* (2021) in this issue of JXB, a further key player in the maintenance of osmotic balance in the symbiosome space of *L. japonicus* nodules has been identified as the methylated polyol, pinitol. Some 13 polyols have been identified in higher plants and they are important antioxidants and osmoprotectants that play key roles in abiotic and biotic stress resistance (Stoop *et al.*, 1996). In *L. japonicus*, the polyols mannitol, pinitol, ononitol (4-*O*-methyl-myo-inositol), threitol, and sorbitol accumulate in nodules (Colebatch *et al.*, 2004; Desbrosses *et al.*, 2005). The production of the methylated derivatives of myo-inositol, either as pinitol or as ononitol, or both by legumes is widespread. Peas produce mainly ononitol and this is probably why some *Rhizobium leguminosarum* strains, as well as *Ensifer meliloti*, produce rhizopines such as 3-OMSI (3-*O*-methyl-scyllo-inosamine), by oxidizing and then aminating ononitol on the 3-*O* group (Geddes *et al.*, 2019). The synthesis of 3-OMSI is under control of the bacteroid-expressed master regulator NifA, and it seems likely that this is then catabolized by sibling rhizobia, either in infection threads or in the rhizosphere. However, the reason for the abundance of pinitol and ononitol in legumes nodules has not been clear until the current work (Tian *et al.*, 2021) which reveals that they are critical to osmotic balance. The authors show by functional analysis in yeast of LjPLT11 from the *L. japonicus–Mesorhizobium* symbiosis that LjPLT11 is an energy-independent transporter for xylitol, two *O*-methyl inositols (pinitol and ononitol), xylose, and galactose. LjPLT11 was shown by immunogold analysis to be located on the symbiosome membrane, where it is predicted to facilitate transport of d-pinitol into the symbiosome space. Knockdown of LjPLT11 by RNAi in *L. japonicus* inhibited plant growth under symbiotic N_2_-fixing conditions and resulted in the formation of abnormal bacteroids with reduced nitrogenase. Strangely, it accelerated plant growth under nitrogen sufficiency, suggesting that synthesis and movement of pinitol has a cost under non-nodulating nitrogen-replete conditions. Interestingly, while LjPLT11 is highly expressed in nodules, it is also expressed in tissues of roots and stems, suggesting that it may have other roles in plant osmotic balance. As predicted from the location of LjPLT11 on the symbiosome membrane, nodules had an increased osmotic pressure in the cytosol and a decreased osmotic pressure in bacteroids particularly 4 weeks after inoculation with *M. loti*. As the symbiosome space is between the bacterial and plant-derived symbiosome membranes, maintenance of the correct osmotic balance would be critical for nutrient exchange, both for uptake of carbon and ions by bacteroids and for secretion of ammonia and alanine to the plant cytosol. It is not clear whether bacteroids themselves accumulate pinitol; however, disruption of the osmotic potential in the symbiosome space is likely to have a knock-on effect on bacteroids. Notably, rhizobia possess their own ways to cope with hyperosmotic conditions. Biosynthesis of compatible solutes such as trehalose or *N*-acetylglutaminylglutamine amide (NAGGN) contributes to adaptation to high osmolarity environments in *Sinorhizobium* (Flechard *et al.*, 2010; Sagot *et al.*, 2010), *Rhizobium* (McIntyre *et al.*, 2007), or *Bradyrhizobium* (Ledermann *et al.*, 2021*a*). Whilst trehalose biosynthesis is needed in *Bradyrhizobium* for growth in infection threads (Ledermann et al., 2018, 2021*a*), no mutants in compatible solute biosynthesis with an effect on bacteroids have so far been reported. This raises the question of how rhizobia adapt to the osmotic conditions in nodules. Bacteroids display a streamlined physiology for N_2_ fixation (Ledermann *et al.*, 2021*b*) and rely on their plant hosts not only for nutrients but also for functions they are able to fulfil as free-living bacteria such as branched-chain amino acid biosynthesis (Prell *et al.*, 2009). The osmoprotective role of d-pinitol and potentially other methylated myo-inositol derivatives transported into the peribacteroid space as described in the study by Tian *et al.* (2021) thus raises the possibility that bacteroids also surrender osmoregulation to their plant hosts. Consistent with the alteration of the osmotic potential in the symbiosome space, RNAi lines of LjPLT11 had misshapen symbiosome membranes. Subsequently, the accumulation and distribution of ROS in infected cells changed in 4-week-old nodules in LjPLT11i plants. It is proposed that this increase in ROS results from the imbalance in osmotic regulation. Overall, this work shows that pinitol maintains osmotic balance and stabilizes the symbiosome membrane.
